# Effect of Amelogenin Solution in the Microhardness of Remineralized Enamel and Shear Bond Strength of Orthodontic Brackets

**DOI:** 10.1155/2021/7025910

**Published:** 2021-10-31

**Authors:** Guilherme Genovez-Júnior, Sandrine Bittencourt Berger, Lucineide Lima dos Santos, Eloisa Aparecida Carlesse Paloco, Murilo Baena Lopes, Débora Fernandes Giuliangeli, Júlia Graciela Monteiro dos Santos, Ricardo Danil Guiraldo

**Affiliations:** Department of Restorative Dentistry, School of Dentistry, University Pitagoras UNOPAR, Londrina, Paraná, Brazil

## Abstract

**Objectives:**

To evaluate the microhardness of tooth enamel remineralized with enamel matrix protein solution as well as the shear bond strength of orthodontic brackets bonded to this surface.

**Materials and Methods:**

In total, 24 human premolars were selected and divided into 3 experimental groups (*n* = 8): SE—sound enamel, DE—demineralized enamel, and TE—demineralized enamel treated with amelogenin solution. Samples from DE and TE groups were subjected to pH cycling to induce initial artificial caries lesion. TE group was treated with amelogenin solution. Samples were placed in artificial saliva for 7 days. Knoop microhardness was measured before any intervention (*T*0), after pH cycling (*T*1) and after amelogenin solution treatment application (*T*2). Twenty-four hours after ceramic orthodontic brackets were bonded, samples were subjected to shear test in a universal testing machine. Microhardness and shear measurement distributions were subjected to Kolmogorov–Smirnov normality test, which was followed by parametric tests (*α* = 0.05): 2-way analysis of variance (factors: enamel condition × treatment) and Tukey posttest for all three groups (SE, DE, and TE) in *T*0 and *T*2 for microhardness; analysis of variance and Tukey's test, for shear bond strength test.

**Results:**

Means recorded for Knoop microhardness in *T*2, for the SE (366.7 KHN) and TE (342.8 KHN) groups, were significantly higher than those recorded for the DE group (263.5 KHN). The shear bond strength of the SE (15.44 MPa) and TE (14.84 MPa) groups statistically differed from that of the DE group (11.95 MPa).

**Conclusion:**

In vitro demineralized enamel treatment with amelogenin solution was capable of taking samples' hardness back to levels similar to those observed for sound enamel. The shear bond strength on the enamel subjected to this treatment was similar to that observed for healthy enamel and higher than that observed for demineralized enamel.

## 1. Introduction

Fixed orthodontic treatments face major challenges during their execution: bracket debonding often takes place, mainly right after bonding or at more advanced stages, when these accessories have already undergone some mechanical and thermal action [1]. In addition, the enamel around the glued accessories undergoes demineralization quite often due to hygiene-associated difficulty faced by patients [2, 3]. The orthodontics of fixed appliances depends on the quality adhesion of its accessories to patients' tooth enamel and, therefore, on the quality of this surface. Thus, the quality of bonding carried out on demineralized enamel is often compromised, which is the reason why orthodontists often need to rebind accessories on this surface [1–3].

Biomimetic strategies have been explored to restore demineralized enamel, and remineralizing agents has been proposed for the treatment of enamel demineralization. In addition to conventional fluoride-based therapies [4], casein phosphopeptide-amorphous calcium phosphate [5], biomimetic hydroxyapatite [6], and peptide-based systems have been introduced recently showing promising results. Nevertheless, the use of amelogenin-based solutions is a promissory strategy adopted to reestablish losses in the enamel matrix framework [7], by allowing mineral nucleation and guiding apatite crystallization in this tissue [7–9]. The amelogenin molecule can be arranged in three portions, namely, central domain, C-terminus (COOH), and N-terminus (NH_2_). C-terminus acts on protein-mineral associations, whereas N-terminus acts on protein-protein associations [10]. Thus, amelogenin-free molecules bind to exposed enamel matrix proteins [11] to form a protein network that will be used as a framework for mineral deposition [7–12]. Amelogenin molecules are grouped into oligomers that, in turn, organize themselves into nanospheres, which are arranged in a “ribbon” to form the framework that will determine the parallelism between crystals [10, 13]. Then, the free minerals' nucleation process in the organic matrix starts to enable hydroxyapatite crystals to grow [10, 11, 13].

Studies were carried out to evaluate the physical-mechanical properties of enamel remineralized with amelogenins; results have indicated amelogenins' likely clinical applications to reverse white spots [14–16] and dental erosion lesions [17], as well as to improve tooth enamel resistance to future acid challenges [18, 19]. Thus, there is urgent need to quantitatively assess adhesion to remineralized enamel based on this strategy. Thus, the aim of the current study was to evaluate the microhardness of dental enamel remineralized with protein solution deriving from the enamel matrix, as well as the shear strength of brackets bonded on this surface.

## 2. Materials and Methods

The present research was submitted for consideration and approval by the Research Ethics Committee of Universidade Norte do Paraná (3,082,100). All teeth used in the study were extracted due to orthodontic indication and donated after patients signed the informed consent form. Sample size was calculated based on the results of a previous study [20] which recorded shear bond strength of human premolars, which had a standard deviation of 4.0 by taking into account the minimum detectable difference of 17.5 on average. Thus, the minimum sampling estimation was considering 3 samples.

### 2.1. Sample Preparation

Twenty-four healthy human premolars were selected and kept at 6°C, right after their extraction, for up to three months. Before any intervention, samples were disinfected with 5% chloramine solution at room temperature, protected from light for 5 days, and stored in distilled water at 4°C [21], until the beginning of the study.

The buccal surface of each tooth was the target of the current study. Thus, teeth were adapted to polyvinyl chloride tubes (Tigre, Castro, PR, Brazil), and their buccal surface was parallel to the long axis of the matrix, so that roots could be included in acrylic resin (Jet Classico, São Paulo, SP, Brazil). An accessory was also made of acrylic resin, which worked as support for the lingual cusp and as stabilizer for the polyvinyl chloride tube, during the microhardness test.

All samples had their microhardness evaluated at three different points in a microhardness tester (HMV-G; Shimadzu, Kyoto, Japan). Results recorded for the initial microhardness test were tabulated, and samples were randomly divided into three experimental groups with 8 samples (*n* = 8) according to treatment: the SE group (sound enamel, untreated) was kept under refrigeration in humid environment (RH 100%), and the DE (demineralized enamel) and TE groups (demineralized enamel, treated with emdogain enamel matrix protein solution (Straumann AG, Basel, Switzerland), which presents as clear gel in a 0.7 ml syringe) were subjected to pH cycling [22].

Enamel demineralization process has followed the protocol proposed by Queiroz et al. [23], based on the pH cycling technique, by alternating 2 hours in demineralizing solution (0.05 mol/L acetate buffer at pH 5.0, 1.28 mmol/L Ca, 0.74 mmol/LP, and 0.3 *µ*g F/mL) and 22 hours in remineralizing solution (0.1 mol/L Buffer Tris at pH 7.0, 1.5 mmol/L Ca, 0.9 mmol/LP, 150 mmol/L KCl, 0.05 *µ*g F/mL) kept at 37°C. The adopted solutions were changed on a daily basis, for 8 days; they were washed by immersion in ultrapure water for 2 minutes, at each change.

Samples from the TE group were subjected to acid etching with 37% acid gel (Villevie, Joinville, SC, Brazil) for 20 seconds, washed with water and air jets for 40 seconds, dried with superficial air jets, and subjected to amelogenin solution application for 15 minutes, according to the previously established methodology [22]. The product was applied in its nondiluted form, in a layer at of least 1 mm (in thickness) that completely covered the exposed tooth enamel surface. Samples from all three groups were then immersed in artificial saliva, at 37°C, for 7 days; the artificial saliva was changed on a daily basis. Artificial saliva solution was prepared based on the formulation proposed by Schimidlin et al. [14].

Again, samples were subjected to microhardness test, at three points (each sample); mean individual microhardness of each sample and mean microhardness of each group were calculated.

### 2.2. Microhardness Test

Each sample was subjected to microhardness testing (HMV-G; Shimadzu, Kyoto, Japan) equipped with a Knoop-type indenter at a static charge of 25 g applied every 5 s, at three different enamel conditions:

- T0—before pH cycling: all samples had their initial microhardness measured at three different points. Results of this test were tabulated; the mean microhardness of each sample and mean microhardness of all samples were calculated.
- T1—after pH cycling: applied to groups 2 and 3, by following the same method described above; this step was only used to monitor the pH cycling results. It was stipulated that cycling would reduce the mean microhardness of the analyzed samples by at least 30%.
- T2—after 7 days of immersion in artificial saliva: applied to all groups.

### 2.3. Brackets' Bonding

Orthodontic brackets' bonding was performed by a single calibrated operator, who initially performed the etching procedure by applying 37% phosphoric acid (Dentsply, Petrópolis, RJ, Brazil), only to the gluing site, with the aid of a syringe, for 30 seconds. Next, the enamel was washed with running water for 30 seconds and dried with light jets of oil-free compressed air for 20 seconds. Subsequently, Transbond XT adhesive primer (3 M ESPE, St. Paul, MN, USA) was applied to the conditioned surface with the aid of disposable microbrush applicator, light cured with Valo (Ultradent, South Jordan, UT, USA) for 10 seconds, in standard mode (395–480 nm; 1000 mW/cm^2^), and a small amount of resin (Transbond; 3 M ESPE) was placed on the base of the Iceram Roth 0.022″ L5A ceramic bracket (Orthometric, Marília, SP, Brazil). The bracket was positioned in the center of the buccal surface of the tooth, with enough pressure to enable the excess material to flow and to be removed with the aid of an exploratory probe. Polymerization was carried out for 40 seconds, 10 seconds on each side of the bracket. Samples were then stored in distilled/deionized water at 37°C for 24 hours.

### 2.4. Shear Bond Strength Test and Failure Analysis

Samples were fitted in a cylinder with jaws; their position was adjusted, so they received the force parallel to the buccal surface of the teeth. The shear knife was positioned at the bracket/enamel interface and subjected to the testing machine (EMIC DL 2000, Equipment and Assay Systems, São José dos Pinhais, PR, Brazil), which was regulated at a speed of 0.5 mm/min until the brackets were removed [24]. Recorded values were converted into MPa, and debonding (kgF) was determined based on the bracket base area informed by the manufacturer (0.12428 cm^2^). Finally, the adhesive remnant index (ARI) was applied to quantify the failure types observed in the samples, based on visual analysis applied to them with the aid of an optical microscope (Eclipse E100; Nikon, Tokyo, Japan) at 40x magnification. This index classifies failures into scores, according to the amount of cementing material that remains adhered to the tooth after debonding, as follows: 0, no remaining material; 1, less than half of the remaining material; 2, more than half of the remaining material; and 3, the whole material remained adhered to the tooth surface and showed the bracket base mesh impression [25].

### 2.5. Statistical Analysis

Statistical analysis was performed in Minitab 16 software for Windows 8 (Minitab, State College, PA, USA). Microhardness and shear measurement distributions were subjected to Kolmogorov–Smirnov normality test, which was followed by parametric tests at 5% significance level (*α* = 0.05): 2-way analysis of variance (ANOVA; factors: enamel condition *x* treatment) and Tukey posttest for all three groups (SE, DE, and TE) in *T*0 and *T*2 for microhardness; analysis of variance (ANOVA) and Tukey's test, for shear bond strength test. The adhesive remnant index was subsequently subjected to descriptive analysis by percentage (%).

## 3. Results

In *T*1, groups DE and TE have lost 34% and 32% of their initial microhardness after pH cycling, respectively. Knoop microhardness data are presented in Table 1. There was interaction between enamel condition and treatment factors (*p*=0.003). Mean Knoop microhardness at three groups denoted by *T*0 ranged from 361.6 to 376.0 KHN with no statistical difference between them; and in *T*2, samples from the TE group presented recovered microhardness; in DE group, this fact did not occur with a statistical difference between enamel conditions *T*0 and *T*2.

**Figure 1:**
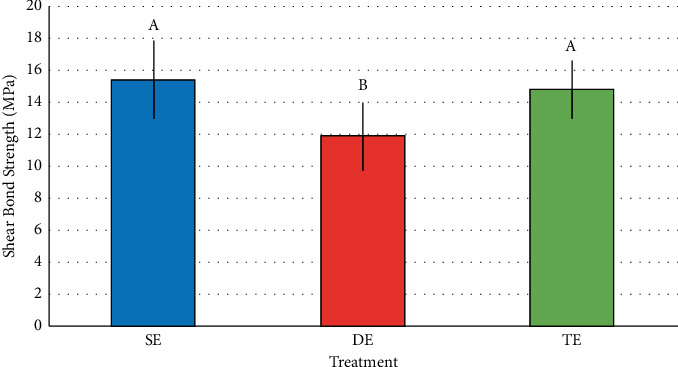
Mean values followed by different uppercase letters differed statistically by Tukey's test at 5% level of significance. SE group: sound enamel, untreated. DE group: demineralized enamel. TE group: demineralized enamel, treated with enamel matrix protein solution.

There was a statistical difference among different groups (*p*=0.009). Mean shear bond strength values recorded for the different groups analyzed in the current study ranged from 11.9 to 15.4 MPa. SE and TE groups recorded significantly higher shear bond strength than the DE group, as shown in Figure 1.

The ARI scores are shown in Table 2. The ARI scores were predominantly 2 e 3 for the SE and TE groups, while for the DE group was predominantly 0.

## 4. Discussion

Concerning to other possibilities for the enamel regeneration, some authors point out biomimetic systems and fluoride boosters have a promising future in dentistry [9]. Biomimetic systems include, in addition to amelogenin based systems, peptide-based systems, the use of poly (amidoamine) dendrimers, nanohydroxyapatite, and electrically accelerated remineralization [9]. Amelogenin and peptide strategies are the more explored on literature, probably due to their capacity to recompose enamel's matrix to guide prism regrowth [10]. Fluoride boosters tend to increase enamel intake of calcium, phosphorous, and fluoride in regular remineralization mechanism, through calcium-phosphate, polyphosphate, or natural products [9]. Some research studies [16, 26, 27] combine both strategies, adding chitosan to amelogenin solution. Other minimally invasive strategies [28, 29] such as infiltrating this tissue with very low viscosity resins are used to manage the white spot lesion, and this would completely stop its progression but without prism regrowth. The present study chose to describe the properties of enamel treated only with amelogenin solution to improve the knowledge about intentionally regrown enamel prisms over a premade protein scaffold.

Results recorded for hardness control during samples' treatment at *T*0 and *T*2 are in compliance with previous studies [22], which used the same sample treatment methodology. TE group has shown Knoop hardness recovery at *T*2, and it indicated successful remineralization of samples subjected to treatment with amelogenin solution. Hardness recovery in the TE group has reached levels similar to the initial ones, although DE, which was not subjected to treatment with amelogenin solution, did not reach this result. Remineralization deriving from amelogenin solution application can be attributed to successful assembly of the new protein scaffold, to the consequent apatite crystallization in this scaffold, and to restore the lost enamel prism volume [7]. Enamel surface featuring through hardness test is often adopted in this type of study to check enamel mineralization. It can be easily performed at each method stage, and it does not compromise or damage samples subjected to shear test [14, 17, 18, 22, 26].

Orthodontic treatment effectiveness depends on accessories' permanence over enamel during force application; there is consensus among scholars about bonding durability in demineralized regions [1, 3, 29]. Studies have shown that accessories' bonding on this type of surface is impaired by molecular changes in apatite crystals [2], as well as by loss of crystal volume and defects in prisms structure. Mean shear bond strength of orthodontic brackets to enamel belonging to different groups ranged from 11.9 to 15.4 MPa in the current study. SE (15.4 MPa) and TE (14.8 MPa) groups statistically differed from the DE (11.9 MPa) group. An important factor to be taken into consideration lies on whether the mean bond strength values are within the range clinically acceptable for orthodontic treatment. However, the literature is not clear about the proper minimum shear bond strength value to be adopted. Based on reports, this value should range from 13.0 to 21.0 MPa [19]; however, other studies [30] reported that it should stay between 6.0 and 8.0 MPa. Based on Zeppieri et al. [19], only the shear bond strengths of the SE and TE groups would be clinically acceptable. However, the current study has shown that, based on the shear bond strength, the remineralizer took the carious enamel back to its initial condition.

ARI evaluation attributed score 2 or 3 to most samples in the SE and TE groups, where most or all the remaining cementing material remained adhered to the tooth. This outcome has evidenced that the bond strength to the enamel was higher than that observed for the retention of this material to the bracket base. On the other hand, most samples in the DE group scored 0 since most or all the remaining cementitious material was adhered to the bracket, which indicated that resin cement adhesion to the bracket mesh surpassed the one observed in demineralized enamel. This finding is in compliance with the literature, which points out that higher ARI scores are indicative of greater adhesive strength to the tooth surface [31]. ARI results corroborated the shear test results, which indicated lower shear strengths in the DE group. Factors such as the bond between the cementitious material and the ceramic, as well as the mesh pattern of the bracket base (squared for this model), could somehow affect the current results. However, if one takes into consideration that all groups were subjected to the same treatment, differences between groups are worth mentioning. Future studies should be carried out in order to assess the influence of the bracket base material, as well as the base mesh/pattern/texture applied in this methodology.

Previous study revealed that 85% of patients who are starting their orthodontic treatment are suffering of at least one enamel demineralized white spot lesion [32]. These lesions may progress rapidly to form enamel and dentin cavities [33] after bonding the orthodontic fixed appliances because of the accumulation of the bacterial biofilm around these appliances [34]. Thus, the remineralization of demineralized enamel prior to bonding is needed. This fact can be obtained by fluoride bioactive glass paste [35] or 45S5 Bioglass [36] that improve bond durability and remineralizes tooth. Another strategy for enamel remineralization is based on peptides that consist of assembling proteins directly in the enamel matrix, whereas the strategy based on amelogenins delivers ready-made proteins to this matrix. The application of amorphous calcium phosphate in association with both techniques appears to have met the mineral demand for apatite prism crystallization in both materials. The main advantage of the amelogenin strategy lies on the fact that the remineralized enamel is more resistant to future acid challenges [18] and, therefore, it would act for longer during orthodontic treatments and likely improve adhesion in future rebonding processes. On the other hand, the risk of debonding after rebonding procedure application to compromised enamel will be even higher. The white spot reversal in the current study was clinically visible and compatible to the remineralization observed at the time enamel hardness was analyzed.

This study, as any in vitro one, has limitations. Regarding its applicability, it might be challenging to reproduce the technique in an in vivo condition, due to the time needed for the remineralization of the new protein matrix: diet texture, mouth temperature, and pH would quickly destroy the exposed scaffold before complete crystal regrowth. This explains why some authors combine a fluoride booster to their solutions. However, the results show a promising future to this strategy as a direct agent of remineralization or as a part of new methods to be developed. Despite the promising results, the literature still lacks further studies about the adhesive behavior of dental materials to remineralized enamel with the aid of enamel matrix proteins. Furthermore, similar to the current study, few articles available in the literature were performed in vitro; therefore, they presented limitations typical of this type of research [37, 38]. The eventual implementation of this technique in situ still faces challenges, mainly when it comes to protect the protein scaffold during apatite crystals' mineralization in the regenerated matrix; however, some strategies appear to be a promising alternative to solve this problem [16, 28, 29]. Therefore, it is necessary performing further studies focused on evaluating the remineralizing capacity of amelogenin solutions in association with faster ion banks, such as chitosan or amorphous calcium phosphate solution, in order to reduce the crystallization time and, thus, enable the clinical applicability of this technique.

## 5. Conclusion

In vitro demineralized enamel treatment with amelogenin solution was capable of taking samples' hardness back to levels similar to those observed for sound enamel. The shear bond strength of enamel subjected to this treatment was similar to that observed for sound enamel and higher than that observed for demineralized enamel.

## Figures and Tables

**Table 1:** Mean knoop microhardness (KHN) results recorded for all three treatment at *T*0 and *T*2.

Enamel condition	Treatment		
	SE	DE	TE
*T*0	376.0 (32.2) Aa	361.6 (27.7) Aa	363.7 (33.6) Aa
	342^*∗*^ 372^*∗∗*^ 438^*∗∗∗*^	326^*∗*^ 364^*∗∗*^ 396^*∗∗∗*^	322^*∗*^ 360^*∗∗*^ 428^*∗∗∗*^

*T*2	366.9 (44.7) Aa	263.6 (49.9) Bb	342.8 (30.9) Aa
	319^*∗*^ 353^*∗∗*^ 442^*∗∗∗*^	68^*∗*^ 270^*∗∗*^ 345^*∗∗∗*^	300^*∗*^ 338^*∗∗*^ 391^*∗∗∗*^

Mean values followed by different lowercase letters in rows and uppercase letters in columns differed statistically by Tukey's test at 5% level of significance. Standard deviations are provided in parentheses. ^*∗*^Minimum value. ^*∗∗*^Median. ^*∗∗∗*^Maximum value. *T*0: enamel before pH cycling. *T*2: enamel after 7 days of immersion in artificial saliva. SE group: sound enamel, untreated. DE group: demineralized enamel. TE group: demineralized enamel, treated with enamel matrix protein solution.

**Table 2:** Frequency distributions of the adhesive remnant index (ARI) scores (%).

Treatment	ARI scores			
	0	1	2	3
SE	0	0	37.5	72.5
DE	50	12.5	12.5	25
TE	0	25	25	50

SE group: sound enamel, untreated. DE group: demineralized enamel. TE group: demineralized enamel, treated with enamel matrix protein solution.

## Data Availability

The data used to support the findings of this study are included within the paper.
